# PReS-FINAL-2101: Nitrous oxide analgesia for intra-articular injection in juvenile idiopathic arthritis: our experience

**DOI:** 10.1186/1546-0096-11-S2-P113

**Published:** 2013-12-05

**Authors:** S Pastore, V Moressa, G Gortani, L Lepore

**Affiliations:** 1 University of Trieste, Trieste, Italy; 2 Institute for Maternal and Child Health - IRCCS "Burlo Garofolo", Trieste, Italy

## Introduction

Nitrous Oxide (NO), known as ''laughing gas'', is a volatile gas with analgesic, anxiolytic and sedative properties, used for treatment of short-lived mild or moderate pain.

## Objectives

To evaluate the efficacy and safety of nitrous oxide-oxygen for children with juvenile idiopathic arthritis (JIA) undergoing intra-articular corticosteroid injection.

## Methods

A 50:50 mixture with NO and oxygen was administered to JIA patients over the age of 5 years scheduled for joint injection. In some cases additional sedative agents (local EMLA, orally midazolam, nasal fentanest) was administered. Every patient completed visual-analogue scores (VAS) (0-10) for pain immediately after the procedure, and after 30 and 60 minutes. The physician valuated sedation level according to Ramsay scale and memory level of the procedure.

## Results

A total of 31 joints were injected in 25 patients (23 F, 2 M, median age 10.4 years). EMLA was placed in all patients at least one hour before the procedure. 19/25 patients received oral midazolam (0.5 mg/kg) 30 minutes before the intra-articular injection. 1/25 patients received nasal fentanest (75γ) during the procedure. The median pain score for patient (0-10 cm VAS) was 0.7 immediately after the procedure, 0.6 after 30 minutes, and 0.5 after 60 minutes. Only 3 out of 25 patients remembered the procedure. There were no adverse events in any patient.

## Conclusion

Nitrous oxide-oxygen provides safe and effective analgesia for JIA children undergoing intra-articular injections, avoiding intravenous cannulation and general anaesthesia.

## Disclosure of interest

None declared.

